# Biallelic inactivation of the retinoblastoma gene results in transformation of chronic myelomonocytic leukemia to a blastic plasmacytoid dendritic cell neoplasm: shared clonal origins of two aggressive neoplasms

**DOI:** 10.1038/s41408-018-0120-5

**Published:** 2018-08-22

**Authors:** Mrinal M. Patnaik, Terra Lasho, Matthew Howard, Christy Finke, Rhett L. Ketterling, Aref Al-Kali, Animesh Pardanani, Nathalie Droin, Naseema Gangat, Ayalew Tefferi, Eric Solary

**Affiliations:** 1 Department of Internal Medicine, Division of Hematology, Mayo Clinic, Rochester, MN USA; 2 Department of Laboratory Medicine and Pathology, Mayo Clinic, Rochester, MN USA; 3 Institute Gustave Roussy, Paris, France

To the Editor,

Chronic myelomonocytic leukemia (CMML) is a clonal hematopoietic stem cell disorder characterized by sustained peripheral blood (PB) monocytosis, bone marrow (BM) dysplasia, and an inherent risk for transformation to acute myeloid leukemia (AML); ~20–30% over 3–5 years^1,2^. Patients with CMML have ~10–15 gene mutations in coding DNA regions, with common mutations involving *TET2* (~60%), *ASXL1* (~40%), *SRSF2* (~40%), and the oncogenic *RAS* pathway (~30%)^3^. Blastic plasmacytoid dendritic cell neoplasm (BPDCN) is a rare hematodermic malignancy of dendritic cell origin that is known to infiltrate the blood, BM, skin, and lymph nodes; a diagnosis of which is based on a blast immunophenotype characterized by the expression of CD4, CD43, CD56, CD123, BDCA-2/CD303, TCL1, and CTLA^1^. Cytogenetic studies, array-based comparative genomic hybridization, and gene expression profiling have demonstrated recurrent inactivation/losses/decreased expression of *RB1* (retinoblastoma 1; 13q13-q21), *LATS2*, *CDKN1B*, *CDKN2A*, and *TP53* genes,^4^ while next-generation sequencing (NGS) studies have demonstrated point mutations involving *TET2* (~36%), ASXL1 (~32%), NRAS (~30%), *ATM* (21%), along with deletions involving *RB1* and *APC* (~6%)^5,6^. In addition, recurrent *MYB* gene rearrangements have been documented in both children and adults, with fusion oncogenes including *MYB-ZFAT*, *MYB-PLEKHO1*, and *MYB-DCPS*^7^. Common clonal origins for CMML and BPDCN have been suggested based on similar genetic and epigenetic deregulations identified, along with sporadic case reports of CMML transformation to BPDCN^8,9^. We provide the first in-depth whole-exome sequencing (WES) results on a patient with CMML that transformed to BPDCN and provide information with regards to possible mechanisms of transformation.

A 61-year-old male was referred to the hematology clinic in 2014 for sustained PB monocytosis. A BM biopsy was suggestive of CMML-0 (World Health Organization 2016 criteria) with normal cytogenetics (Fig. 1). He did have plasmacytoid dendritic cell nodules comprising ~20% of the BM cellularity. NGS at diagnosis identified mutations involving *TET2* (variant allele frequency (VAF) 40%) and *SRSF2* (43%). He was conservatively managed with routine blood count checks. In 2017, he presented with worsening constitutional symptoms, a generalized skin rash, and progressive cytopenias. A BM biopsy and skin biopsy were suggestive of BPDCN with normal BM metaphase cytogenetics (Fig. 1). He was treated with AML-like induction chemotherapy using idarubicin and cytarabine and then received salvage chemotherapy with fludarabine, idarubicin, and cytarabine, but was found to have persistent disease and died secondary to infectious complications. BM DNA was available at the time of CMML diagnosis and at transformation to BPDCN. In addition, sorted T lymphocytes at CMML diagnosis were isolated and cultured to serve as a potential germline control for WES.

**Fig. 1:**
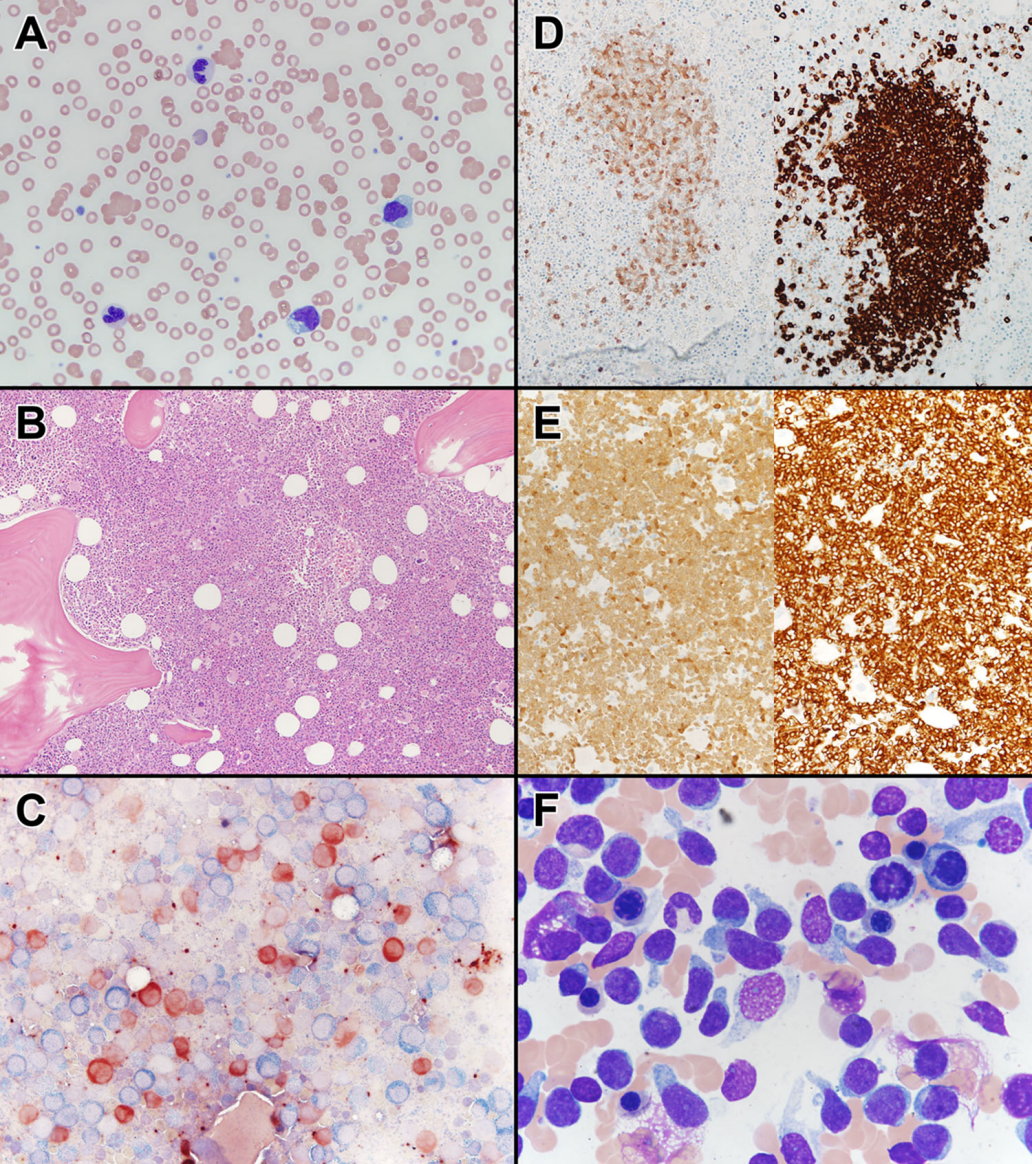
**a** Peripheral blood smear of the patient with a diagnosis of chronic myelomonocytic leukemia, demonstrating atypical dysplastic granulocytes, atypical monocytes, and a blast. Wright Giemsa stain, ×400 magnification. **b** Bone marrow core biopsy of the patient with chronic myelomonocytic leukemia, demonstrating a hypercellular marrow (90%), with dysplastic megakaryocytes. Hematoxylin and eosin, ×100 magnification. **c** Butyrate esterase (brown) and chloroacetate esterase (blue) cytochemical dual stain, demonstrating increased butyrate esterase monocytes and dual esterase-positive bone marrow monocytes, suggestive of monocytic dysplasia (×400 magnification). **d** (Left) Bone marrow core biopsy, TCL1 immunohistochemistry, demonstrating TCL1-positive plasmacytoid dendritic cell nodules (×200 magnification). (Right) Bone marrow core biopsy, CD123 immunohistochemistry, demonstrating CD123-positive plasmacytoid dendritic cell nodules (×200 magnification). **e** (Left) Bone marrow biopsy, TCL1 immunohistochemistry, demonstrating diffusely TCL1-positive blasts, suggestive of a blastic plasmacytoid dendritic cell neoplasm (×100 magnification). (Right) Bone marrow core biopsy, CD123 immunohistochemistry, demonstrating diffusely positive CD123 blasts, suggestive of a blastic plasmacytoid dendritic cell neoplasm (×100 magnification). **f** Bone marrow aspirate obtained at the time of disease transformation demonstrating hand mirror-shaped blastic cells, characteristic for blastic plasmacytoid dendritic cell neoplasms. Wright Giemsa, ×1000 magnification

Two hundred nanogram of genomic DNA was sheared with the Covaris S2 system (LGC Genomics/KBioscience). DNA fragments were end repaired, extended with an “A” base on the 3′ end, ligated with paired-end adaptors with the Bravo Platform (Agilent), and amplified (six cycles). Exome-containing adaptor-ligated libraries were hybridized for 40 h with biotinylated oligo RNA baits, and enriched with streptavidin-conjugated magnetic beads using SureSelect Clinical Research (Agilent). The final libraries were indexed, pooled, and sequenced on Illumina HiSeq-2000 sequencer at the Institute Gustave Roussy (Paris, France). Raw reads in FASTQ format from each exome sequencing lane were aligned to the reference human genome (Hg19) using Burrows Wheeler-Alignment. Aligned reads were processed and sorted with SAMtools and PCR duplicates were removed. Nucleotide variants (single-nucleotide polymorphisms and indels) were called with VarScan and all variants with a Phred-based quality score <30.0 were called low quality and ignored. On an average, 369 million reads were sequenced per sample. A formal copy number variation (CNV) analysis was carried out on the WES data, both at CMML diagnosis and at BPDCN transformation, using the FACETS analysis software (https://sites.google.com/site/mskfacets).

WES results on the DNA specimen obtained at CMML diagnosis identified the following pathogenic variants *TET2* (c.1567_1568insA, p.G523Efs*44; VAF 57%), *SRSF2* (c.284C>T, p.P95L; VAF 37%), *PHF6* (c.753G>T, p.Q251H; VAF 57%), *PLCXD3* (c.845C>T, p.T282M, VAF 39%), *TRMT61B* (c.656A>G, p.T219C, VAF 20%), *STK3* (c.618del, p.N207Ifs*3, VAF 42%), *SLC25A10* (c.787C>T, R263C, VAF 32%), *DIP2A* (c.1117C>T, R373W, VAF 35%), *SARDH* (c.1922G>A, p.G641E, VAF 19%), *PLP1* (c.347C>T, T116M, VAF 74%), and *IVL* (c.706_735dup, P236_L245dup) (Fig. 2). At the time of BPDCN transformation, WES demonstrated acquisition of the following mutations: *RB1* (c.751C>T, p.R251*; VAF 68% secondary to loss of heterozygosity of 13.q13-24), *CROCC* (c.4595C>G, T1532S, VAF 44%), *ERCC4* (c.2608G>A, p.V870I, VAF 38%), and *CHP2* (c.101G>A, R34Q, VAF 32%), while mutations involving the following genes were no longer detected, *IVL* and *RFPL1*. An increase in the VAF burdens in the following genes was also encountered at the time of BPDCN transformation, *PHF6* (57–78%), *TET2* (57–77%), *PLCXD3* (39–43%), *TRMT61B* (20–40%), *SLC25A10* (32–41%), *DIP2A* (35–40%), *SARDH* (19–34%), and *PLP1* (74–91%). A formal CNV analysis also confirmed loss of heterozygosity of chromosome 13, involving the *RB1* gene locus (supplemental figure).

**Fig. 2:**
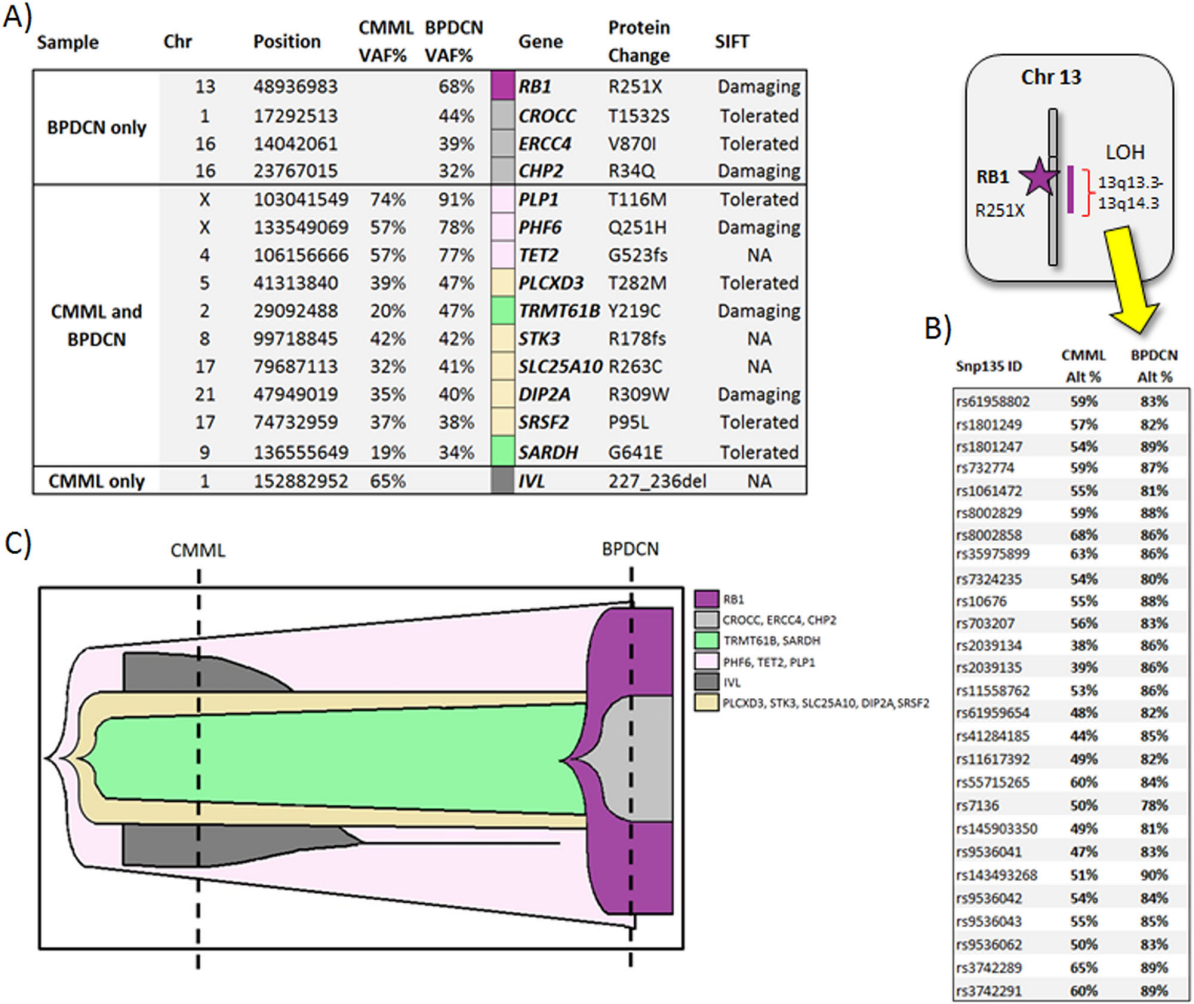
Paired and germline whole-exome sequencing data obtained at the time of chronic myelomonocytic leukemia (CMML) diagnosis and at CMML transformation to a blastic plasmacytoid dendritic cell neoplasm (BPDCN). **a** Paired and germline whole-exome sequencing data obtained at the time of CMML diagnosis and at CMML transformation to BPDCN, demonstrating the biallelic inactivation of the *RB1* gene. **b** Whole-exome sequencing data from chromosome 13, demonstrating biallelic inactivation of the *RB1* gene secondary to loss of heterozygosity and the acquisition of a nonsense *RB1* gene mutation (c.751C>T, p.R251*). **c** SciClone analysis demonstrating the clonal architecture and mutational evolution spectrum in a patient, at CMML diagnosis and at CMML transformation to BPDCN

CMML blast transformation morphologically almost always results in an AML phenotype and occurs due to the clonal acquisition of cytogenetic and molecular abnormalities^2,3,10^. In a large, two-center study of 171 patients with blast phase CMML, all patients met the morphological criteria for a diagnosis of AML (secondary AML) and cytogenetic clonal evolution was seen in 24% of patients, while molecular clonal evolution was seen in 50% of patients^2^. Blast transformation from CMML to BPDCN is an extremely rare event and is usually associated with CNVs and unique molecular changes^9^. In our patient, the main genetic event identified at BPDCN transformation was a truncating mutation in *RB1*, along with loss of heterozygosity of the *RB1* gene, resulting in biallelic inactivation of *RB1* (Fig. 2). The *RB1* gene encodes a protein with tumor suppressor function that serves as a G1 checkpoint inhibitor (by inhibiting E2F transcription factors), a regulator of apoptosis and helps maintain permanent cell cycle arrest and chromosomal stability^11^. In addition, *RB1* also acts as a transcription cofactor and an adaptor protein promoting the function of critical transcription factors^11^. Loss of *RB1* function is associated with progression of human cancers via loss of cellular differentiation and chromosomal instability^11^. While *RB1* mutations and deletions are common in BPDCN, they are infrequent in CMML or secondary AML arising from CMML, supporting our hypothesis that in our patient, biallelic loss of *RB1* maybe a major contributor of a BPDCN phenotypic transformation^3^. Additional genetic events acquired at the time of BPDCN transformation included mutations involving *ERCC4* (Excision Repair 1, Endonuclease Non-Catalytic Subunit) and *CHP2* (Calcineurin B Homologous Protein 2). While these genes have important physiological functions and have been implicated in oncogenesis, their contribution to BPDCN transformation needs further elucidation. Increase in mutational frequencies of genes identified at CMML diagnosis, including *TET2* and *PHF6* are consistent with the process of clonal evolution and disease transformation. Interestingly, the VAF burden of *SRSF2*, a gene regulating pre-mRNA splicing, remained the same at CMML diagnosis and at BPDCN transformation^12^.

By performing WES in paired samples and in the germline of a single patient with BPDCN transformation from an underlying CMML, we demonstrate (i) the common clonal origins of CMML and BPDCN, justifying the current inclusion of BPDCN as a myeloid neoplasm, (ii) the fact that the blast phenotype at CMML transformation is heavily dependent on the nature of genetic changes that occur at blast transformation, iii) the importance of the *RB1* gene in BPDCN morphology and oncogenesis, and (iv) the role of clonal acquisitions and losses and increases in existing mutational allele burdens in disease progression/blast transformation.

## Electronic supplementary material

Supplementary Figure Legend

Supplemental figure
