# Supplementary material for: Biallelic inactivation of the retinoblastoma gene results in transformation of chronic myelomonocytic leukemia to a blastic plasmacytoid dendritic cell neoplasm: shared clonal origins of two aggressive neoplasms
Source: Blood Cancer J. 2018 Aug 22;8(9):82. doi: 10.1038/s41408-018-0120-5 (PMC6127132; doi:10.1038/s41408-018-0120-5)
Supplement: Supplementary file 1 — Supplementary Figure Legend [file 41408_2018_120_MOESM1_ESM.pdf]

Supplementary Material File

Supplemental Figure: Formal copy number variation analysis (CNV) carried out on the WES data set, both, at CMML diagnosis and at BPDCN transformation, using the FACETS analysis software. The top panel for each sample depicts total copy number log-ratio (logR) arranged by chromosome, and the second panel displays allele-specific log-odds-ratio data (logOR) which is an estimate of the allelic copy ratio. The third panel plots the corresponding integer (total -black, minor -red) copy number calls. The arrows denote chromosome 13 and demonstrate loss of heterozygosity of the *RB1* gene locus.
