# Supplementary figures and images for: Biallelic inactivation of the retinoblastoma gene results in transformation of chronic myelomonocytic leukemia to a blastic plasmacytoid dendritic cell neoplasm: shared clonal origins of two aggressive neoplasms
Source: Blood Cancer J. 2018 Aug 22;8(9):82. doi: 10.1038/s41408-018-0120-5 (PMC6127132; doi:10.1038/s41408-018-0120-5)

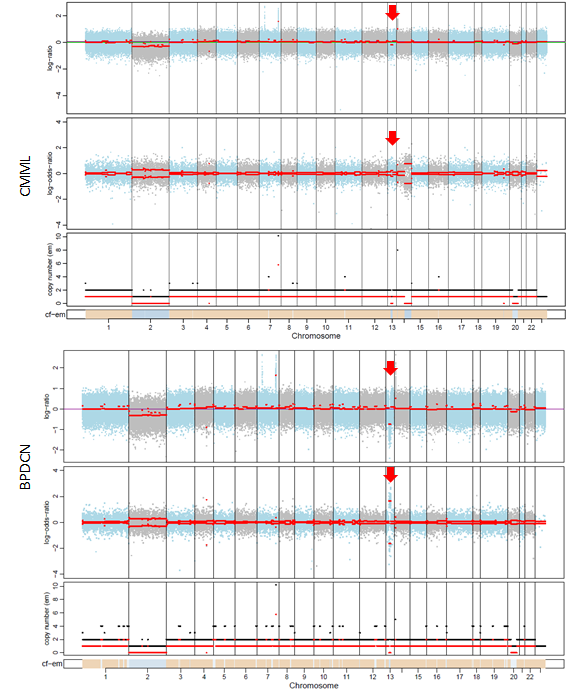

Supplement: Supplementary file 2 — Supplemental figure [file 41408_2018_120_MOESM2_ESM.tif]
